# Accurate segmentation of neonatal brain MRI with deep learning

**DOI:** 10.3389/fninf.2022.1006532

**Published:** 2022-09-28

**Authors:** Leonie Richter, Ahmed E. Fetit

**Affiliations:** ^1^Department of Computing, Imperial College London, London, United Kingdom; ^2^UKRI CDT in Artificial Intelligence for Healthcare, Imperial College London, London, United Kingdom

**Keywords:** semantic segmentation, deep learning, transfer learning, neonates, MRI, label budget

## Abstract

An important step toward delivering an accurate connectome of the human brain is robust segmentation of 3D Magnetic Resonance Imaging (MRI) scans, which is particularly challenging when carried out on perinatal data. In this paper, we present an automated, deep learning-based pipeline for accurate segmentation of tissues from neonatal brain MRI and extend it by introducing an age prediction pathway. A major constraint to using deep learning techniques on developing brain data is the need to collect large numbers of ground truth labels. We therefore also investigate two practical approaches that can help alleviate the problem of label scarcity without loss of segmentation performance. First, we examine the efficiency of different strategies of distributing a limited budget of annotated 2D slices over 3D training images. In the second approach, we compare the segmentation performance of pre-trained models with different strategies of fine-tuning on a small subset of preterm infants. Our results indicate that distributing labels over a larger number of brain scans can improve segmentation performance. We also show that even partial fine-tuning can be superior in performance to a model trained from scratch, highlighting the relevance of transfer learning strategies under conditions of label scarcity. We illustrate our findings on large, publicly available T1- and T2-weighted MRI scans (*n* = 709, range of ages at scan: 26–45 weeks) obtained retrospectively from the *Developing Human Connectome Project (dHCP)* cohort.

## 1. Introduction

How the human brain develops is a fundamental question in neuroscience that motivated initiatives like the *Developing Human Connectome Project (dHCP)* (Makropoulos et al., 2018b) and the *Baby Connectome Project* (Howell et al., 2019) to seek out a blueprint of the developing brain. Mapping out neonatal brain development could help accelerate early diagnosis and detection of diseases such as cerebral palsy, autism, hypoxic ischemic encephalopathy, and congenital deformations, which are thought to originate during the perinatal period of human development (Devi et al., 2015; Makropoulos et al., 2018b). With the help of advanced imaging techniques, it is indeed possible to observe the structural and functional development of the brain in the neonatal developmental phase (Devi et al., 2015). Magnetic Resonance Imaging (MRI) in particular is an excellent source of data, since data acquisition is carried out non-invasively and at high resolution. When an image needs to be further processed, for example to calculate the volume of different brain areas to detect abnormalities (Van Leemput et al., 1999; Makropoulos et al., 2014), the first step is often an image segmentation task, and accurate structural processing of MRI data is also an important step toward delivering an accurate connectome of the developing brain. Automating the segmentation of neonatal brain MRI would therefore be very beneficial for the neuroimaging community.

In image segmentation tasks, deep learning-based algorithms have been at the forefront of development in recent years, including in the broad area of medical imaging (Litjens et al., 2017; Makropoulos et al., 2018a). Common applications include brain imaging [e.g., tumor (Menze et al., 2014) and structure segmentation (Wu and Tang, 2021)], lung, prostate (Litjens et al., 2017), and breast tissue segmentation (Zhang et al., 2020). The U-Net architecture, a model developed specifically for image segmentation which expands on the idea of a fully convolutional network (Ronneberger et al., 2015), has become one of the most widely-used architectures for semantic segmentation applications in the field of biomedical imaging (Litjens et al., 2017; Isensee et al., 2018). This can be seen, for example, in the Kidney Tumor Segmentation Challenge (KiTS), a large-scale challenge with over 100 participants (Isensee et al., 2019), where all models in the top 15 were based on the U-Net architecture. Common architectural modifications such as residual connections or attention mechanisms have not shown a consistent advantage (Isensee et al., 2019). Conversely, even amongst the top 15 in the KiTS leaderboard rankings, there were still unmodified versions of the U-Net architecture to be found. Isensee et al. (2018) therefore argue that the effect of an optimal combination of design choices and hyperparameters is significant. Since finding these optimal hyperparameters can become a challenge, Isensee et al. (2018) proposed a solution to this problem by condensing domain knowledge into heuristics that suggest a model with certain design choices, i.e., “pipeline fingerprint” for a dataset with certain properties compiled in a “data fingerprint,” calling it nnU-Net (“no-new-Net”). The framework has been shown to perform competitively on the Medical Segmentation Decathlon challenge, which consists of 10 different datasets with different modalities (CT, MRI, EM) and mapped organs, e.g., heart, liver, brain, amongst others (Isensee et al., 2019).

Nevertheless, obtaining sufficient ground truth labels for data-driven methods based on deep learning can be expensive, cumbersome, and time-consuming. For neonatal brain MRI, there are further complications associated with the data; for instance, the scans can be very heterogeneous in morphology and texture, which is caused by the rapid brain development taking place over narrow time-scales (Prayer, 2011). Other factors include lower signal-to-noise ratio, lower contrast-to-noise ratio, motion artifacts, and inverted signals. The biological process of myelination, which occurs especially in the time span between midgestation and the end of the second year of life (Sampaio and Truwit, 2001; Branson, 2013), is also of particular importance here. Myelination describes the process by which oligodendrocytes in the central nervous system and Schwann cells in the peripheral nervous system form a myelin sheath around axons (Salzer and Zalc, 2016). This process has various functions, including accelerating the electrical transmission of information in the CNS and providing axonal support, and is involved in learning and memory consolidation (Lazari and Lipp, 2021). White matter, which appears bright in T1-weighted adults due to myelination, appears inverted in infant brain scans with incomplete myelination (Makropoulos et al., 2018b); this difference in brightness can already be seen between preterm and term infants. In this regard, addressing the ground truth bottleneck is essential for deep learning to find its way in neonatal image segmentation tasks.

### 1.1. Contributions

In this paper, we make three novel contributions to the area of neonatal brain MRI segmentation:

*(1)* We present a fully automated, deep learning pipeline for segmenting 3D neonatal brain MRI that achieves high segmentation performance on subjects of a wide age range. We developed and tested the method on a large, publicly available dataset of infant brain scans and their corresponding segmentation labels provided by the *dHCP* initiative. Additionally, we extended the deep learning pipeline's functionality by introducing an age prediction pathway. The code for the deep learning pipeline, as well as all experiments presented in this paper, is publicly available on Github^1^.
*(2)* A major constraint to using deep learning is the need to collect large numbers of ground truth labels. Therefore, in addition to the presented pipeline, we examined different strategies for dealing with the ground truth bottleneck in the context of neonatal MRI. We framed the problem of insufficient ground truth as having a limited budget of labels that needs to be distributed over an unlabeled training set. In other words, given a large dataset of neonatal 3D scans, what would be the most efficient way to generate labels under a constrained budget? A key variable is therefore the extent to which the labels are distributed over a large portion of the subjects *across* the dataset. A second variable concerns the question of how exactly the labels should be distributed *within* a single subject. For example, does it make a difference whether an expert annotates axial 2D slices or sagittal slices? Does it make a difference if one of the axes has a lower resolution than the others? To study this, we made use of the publicly available *dHCP* labels in place of ground truth labels in order to “simulate” different labeling strategies.
*(3)* Lastly, we looked into the extent to which transfer learning can remedy the problem of limited annotated training data. Different strategies of transfer learning were compared to explore how a model trained on a population of older neonates can achieve good segmentation performance on a group of preterm infants, both obtained from the public *dHCP* release.

## 2. Related work

According to Makropoulos et al. (2018a), most of the common segmentation methods for neonatal brain MRI can be divided into five groups: unsupervised methods, parametric methods, classification methods, atlas fusion methods, and deformable models. Application of atlas fusion techniques on their own are rare in the neonatal brain segmentation literature, possibly because their performance is always constrained by the availability of a suitable, age-dependent atlas, as well as by the quality of the registration algorithm (Makropoulos et al., 2018a). They are sometimes used in combination with parametric techniques, e.g., Expectation Maximization (EM) algorithms, which model the distribution of voxel intensities by a posterior probability with a spatial prior term and an intensity term. Parametric techniques are some of the most widely used segmentation methods for neonatal brain MRI because of their robustness to differences in anatomy (Makropoulos et al., 2018a,b). The quality of the initial segmentation is not essential, since it is iteratively improved upon as the algorithm progresses. In classification techniques, the segmentation problem is interpreted as a voxel-wise classification problem and a classifier is trained using data consisting of pairs of MR images and segmentation masks (i.e., atlases). These methods have many advantages: they show high performance and require comparatively less experience and expertise than, for example, developing handcrafted features (Makropoulos et al., 2018a). Classification techniques mentioned here include k-NN, Naive Bayes, Decision Forests, Support Vector Machines (SVMs), and Convolutional Neural Networks (CNNs). More recent approaches include the one by Moeskops et al. (2016). Their approach relied on a multi-scale CNN and voxel-wise classification. To utilize spatial information more efficiently, multiple patch sizes and kernel sizes were used. They tested their method on three neonatal datasets in particular: a set of coronal T2-weighted images of preterm infants acquired at 30 weeks post-conceptional age, a set of T2-weighted images of preterm infants acquired at 40 weeks post-conceptional age as well as axial T2-weighted images of preterm infants acquired at 40 weeks post-conceptional age. The infant brains were segmented into eight classes: Cerebellum, Myelinated White Matter (mWM), Basal Ganglia and Thalami (BGT), Ventricular Cerebrospinal Fluid (vCSF), Unmyelinated White Matter (uWM), Brainstem (BS), Cortical Gray Matter (cGM), and Extracerebral Cerebrospinal Fluid (eCSF). Moeskops et al. (2016) report mean Dice Similarity Coefficient (DSC) values of 0.87, 0.82, and 0.84 on these datasets, respectively. Beyond the choice of segmentation algorithms, other parts of an MRI processing pipeline have to be adapted to the neonatal data. For example, motion artifacts can be prevented by fast scanning techniques or correction algorithms can be applied afterwards to reconstruct a coherent 3D image from misaligned 2D slices. Furthermore, pre-processing steps such as brain extraction and intensity inhomogeneity correction are often applied (Smith, 2002; Makropoulos et al., 2018a).

The standard assumption in machine learning is that training and testing data are drawn from the same feature space and from the same distribution; this assumption can be relaxed in transfer learning (Pan and Yang, 2009). In settings where the training data is scarce, which is often the case for complex biomedical data, using previously generated knowledge in new domains of interest can be beneficial. Additionally, data almost never stems from the same distribution in practice; the modality, the scanner that is used, the acquisition protocol, the hospital and, of course, the underlying population that is investigated all might differ from sample to sample (Van Opbroek et al., 2014). Deep learning is particularly well-suited for transfer learning, since deep convolutional networks are assumed to learn a hierarchy of multiple levels of representation with more abstract, domain-specific features in the higher levels of the representation and more general features in the lower levels (Bengio, 2012; Tajbakhsh et al., 2016). A lot of the early transfer learning experiments used CNNs as off-the-shelf feature generators and fed the CNN features to a separate classifier, usually an SVM or a Decision Forest (Azizpour et al., 2015; Kornblith et al., 2019). Azizpour et al. (2015) identified some of the relevant factors when it comes to the question of how a deep CNN representation should be learned and adjusted to facilitate transfer between source and target task, including the nature of the source task and its distance from the target task, network parameters like width, depth, etc., whether the network is fine-tuned using labeled data from the target task and which layer of the network the representation should be extracted from. They observed that the “optimal settings are clearly correlated with the distance of the target task[...] from the source task” (p. 2). If the target task is very distant from the source task they suggested extracting the representation from earlier layers of the network. This was also shown by Yosinski et al. (2014).

Tajbakhsh et al. (2016) shed light on the division between feature-extraction and fine-tuning variants of transfer learning. While in the first case features from a certain layer of a pre-trained network are extracted and fed to a new classifier, e.g., an SVM, the second approach aims to adapt the pre-trained network to the target task by updating all layers during training. Tajbakhsh et al. (2016) investigated a hybrid approach in which the weights of an AlexNet are initialized with those of an ImageNet pre-trained network and then, starting from a feature extraction approach, more layers are included in the fine-tuning process, while the earlier ones remain fixed. Tajbakhsh et al. (2016) coined the term “shallow fine-tuning” for conditions where only late layers are updated. Conversely, the approach that is usually simply called “fine-tuning” can be referred to as “deep fine-tuning.” There is no consensus yet on whether feature extraction or fine-tuning is generally better, but there is much to suggest that it depends on a number of factors such as the proximity of the source task to the target task and the domain. Moreover, the choice also presents a trade-off where feature extraction is usually associated with less computational effort and a shorter training time (Mormont et al., 2018).

There are several examples of successful application of deep transfer learning strategies to medical computer vision problems. Particularly noteworthy here is the paper by Tajbakhsh et al. (2016) in which the authors investigated four applications (polyp detection in colonoscopy videos, image quality assessment in colonoscopy videos, pulmonary embolism detection, and intima-media boundary segmentation) as well as three different medical imaging modalities (MRI, CT, and ultrasound). In their experiments, they not only examined fine-tuned and feature-extracting models, but also compared them to another identical AlexNet model trained from scratch. They analyzed how the availability of training samples influenced the performance of the different models, lowering the amount of training data to 70%, 50%, and 25% of the original training data. They found that, with deeper fine-tuning, performances comparable and even superior to AlexNets trained from scratch could be achieved; and that the performance gap between deeply fine-tuned networks and those trained from scratch widened when the size of training sets was reduced, i.e., the fine-tuned network was more robust against small training dataset sizes. In most cases, even the (deeply-tuned) model trained only on 25% of the training data still achieved good performance metrics (Tajbakhsh et al., 2016). They also report gains in the speed of convergence for fine-tuned models in comparison to models trained from scratch and conclude stating “fine-tuned CNNs should always be the preferred option regardless of the size of training sets available” (p. 11). How deeply one should fine-tune would then be dependent on the difference between the source domain and the target domain, with the transition from natural images to the medical domain requiring deeper levels of fine-tuning. Mormont et al. (2018) investigated deep transfer learning strategy for digital pathology, comparing a variety of state-of-the-art network architectures and a variety of feature extraction and fine-tuning approaches, suchas extracting information from one specific layer of the source network, or merging features from several layers. They found that fine-tuning usually outperformed all other methods regardless of the network which was used. When feature extracting, they found that last layer features were always outperformed by features taken from an inner layer of the network (which was always located rather at the end of the network).

Although there is a wealth of literature on the application of transfer learning in medical imaging, the majority of these applications relate to classification problems and architectures typically used for that purpose, such as ResNets and DenseNets. For these types of networks, it is easy to give a definition of “shallow” and “deep” tuning, but this becomes more complicated for deep learning based segmentation methods, and especially when it comes to U-Net-like architectures. Amiri et al. (2019) state: “It is important to note that U-Net is not a simple feedforward architecture. The notion of deep and shallow is ambiguous in a U-Net, because there are short and long paths from the input to the output.” (p. 7). The work of Amiri et al. (2019) is also one of the few studies available to date that looked at different ways of fine-tuning the U-Net. They investigated two target segmentation tasks, both in medical imaging: first, segmentation of ultrasound B-mode images of the breast with either benign lesions or malignant tumors, and second, segmentation of lung lobes from chest X-ray. Both datasets consisted of a rather small number of 2D images (163 and 240, respectively). In both cases, the U-Net was first pre-trained on the XPIE dataset with 10,000 natural images (as in the case of the medical data, there was only one foreground and one background class). In a first set of experiments, Amiri et al. (2019) then examined two approaches: one where they only fine-tuned the contracting path of the U-Net, and one where they only fine-tuned the expanding path, leaving the weights of the other part of the network frozen in each case. Here, they found that fine-tuning (only) the contracting path led to better segmentation performance in the case of the ultrasound image dataset, while both approaches produced similar results for the X-ray dataset. In a second part, they investigated the effect of different degrees of shallow and deep fine-tuning. Assuming that earlier layers of a CNN learn more general surface features and that basic features of the natural and the biomedical images are similar, it would be reasonable to start by training the deeper (i.e., later) layers of the U-Net first, and then successively add earlier layers to the training. However, Amiri et al. (2019) argue that since medical imaging methods sometimes produce characteristic, modality-specific artifacts (e.g., speckles caused by wave-tissue interactions), the assumption of surface similarity might be violated. They observed that performance improved when starting with the shallow layers and then including deep layers in the training than when, conversely, the deep layers were fine-tuned first.

## 3. Materials and methods

### 3.1. Dataset

In this paper, we made use of publicly available data provided by the *dHCP*, an initiative that seeks to create “a dynamic map of human brain connectivity from 20 to 44 weeks post-conceptional age (PMA) from healthy, term-born neonates, infants born prematurely (prior to 36 weeks PMA) and fetuses” (Makropoulos et al., 2018b, p. 3). The most recent, publicy available *Third Data Release* includes 783 3D neonatal MRI scans. For the purpose of this study, T1- and T2-weighted MRI scans and minimal meta data were analyzed retrospectively. Corresponding segmentation labels produced by the *dHCP* structural pipeline served as a pseudo ground truth for our purposes. The segmentation labels represented the following classes: Cerebrospinal Fluid (CSF), Cortical Gray Matter (cGM), White Matter (WM), Ventricles, Cerebellum, Deep Gray Matter (dGM), Brainstem, Hippocampus, as well as an inner background class representing regions bordering brain tissues (Makropoulos et al., 2014). Additionally, there was an outer background class representing regions of the image surrounding the skull (pixel intensity values of 0). These segmentation labels were based exclusively on the T2-weighted data, since due to incomplete myelination, the contrast of the T1-weighted scans is inverted (Makropoulos et al., 2018b).

**Figure 1:**
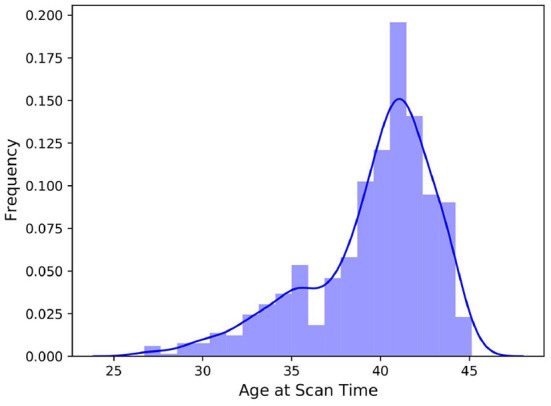
Histogram of the distribution of scan ages in the full *dHCP* dataset consisting of 709 samples. The added kernel density estimate highlights the bimodal distribution of the data.

The 3D scans and segmentation labels were available in NIfTI file format, and all scans had a resolution of 217 × 290 × 290. Not all four file types (T1-weighted, T2-weighted, segmentation labels for all classes, and minimal meta data) were available for all subjects, thus, we excluded incomplete data units which left us with 709 subjects. Figure 1 depicts the negatively skewed distribution of scan ages in the dataset. The median scan age in the sample was 40.57 weeks, while the average scan age was 39.56 weeks. Preterm born infants are usually defined as infants born before 37 weeks of gestational age, which was the case for 148 subjects in the dataset. The youngest subject was scanned at 26.71 weeks post-conceptional age while the oldest was 45.14 weeks post-conceptional age at time of scan.

### 3.2. Pipeline design

U-Net-like architectures represent the state-of-the-art in medical image segmentation (Isensee et al., 2018; Cai et al., 2020) and show excellent performance in a variety of tasks (Isensee et al., 2019), making the use of U-Net a natural starting point. As described by Isensee et al. (2018)), parameters of the pipeline such as pre-processing steps, exact network topology, and the selection of an appropriate loss function, among others, can have significant effects on performance and must be determined carefully. Isensee et al. (2019) analyzed the interdependencies between the dataset fingerprint and the pipeline fingerprint and divided the pipeline parameters into fixed parameters, rule-based parameters, and empirical parameters. In constructing our pipeline, we followed their recommendations and rules to a large extent.

#### 3.2.1. Pre-processing

To increase information density and save computation power, training, and validation images were first cropped to the region of non-zero values (while still keeping a cuboid shape) using a bounding box. Images were then cropped into patches. In general, the patch size should be as large as possible to allow the network to include as much context information as possible (Isensee et al., 2018). However, GPU memory also exerts constraints on patch size, especially if one considers that a batch size of two should not be undercut in training in order to avoid instabilities. Lastly, the network architecture (see section 3.2.4) also made special demands on the size of the input patches. In particular, the patch size along each of the three dimensions must be divisible by $2^{n_{d}}$, where *n*_*d*_ is the number of downsampling operations. The highest patch size compliant with all these constraints, 128 × 128 × 128, was chosen. Following Isensee et al. (2019)'s recommendation, resampling was omitted. Subject scans were individually intensity-normalized, on a channel-wise basis.

#### 3.2.2. Data augmentation

**Figure 2:**
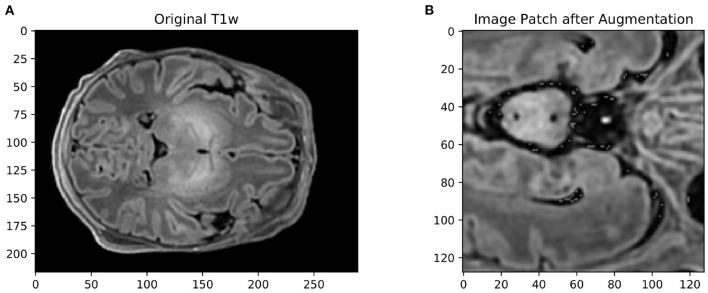
**(A)** Example of an axial slice from a T1-weighted scan in the dataset, and **(B)** the same image after data augmentation The size of the original image was 217 × 290 × 290. The augmented image was first cropped to the size of the brain, then randomly cropped to 128 × 128 × 128, mirrored, elastically deformed, Gaussian noise and Gaussian smoothing added, and then voxel intensity scaled and shifted.

Isensee et al. (2018) showed that omitting data augmentation was the only pipeline parameter that consistently degraded performance across all medical imaging datasets they examined. Data augmentation is often a means of choice when training data is scarce, as it allows for the constant generation of “new” training data. It can also have a regularizing effect on the data and help prevent overfitting. We therefore attempted to cover a wide range of spatial and intensity-based transformations such as random spatial cropping, flipping, 3D elastic deformation, Gaussian noise, Gaussian smoothing, intensity scaling, and intensity shifting. All transformations were applied probabilistically, ensuring that the network still encountered unaltered training images with sufficient frequency. An example of an augmented patch can be seen in Figure 2. Cirillo et al. (2021) showed that elastic deformation augmentation in particular were very realistic and led to better performance in tumor segmentation, probably because tumors are subject to large degrees of spatial variation; this is also typical of neonatal brains.

#### 3.2.3. Training hyperparameters

Following Isensee et al. (2018), a combined loss of Dice Loss and Cross Entropy Loss was chosen:


(1) $$\begin{array}{l} L_{\text{total}}=L_{\text{Dice}}+L_{\text{CE}} \end{array}$$

The Dice Loss, which is derived from the Dice Similarity Coefficient (DSC), was calculated according to the formula (Isensee et al., 2018):


(2) $$\begin{array}{l} L_{\text{Dice}}=-\frac{2}{\left|K\right|}\sum_{k\in K}\frac{\sum_{i\in I}u_{i}^{k}v_{i}^{k}}{\sum_{i\in I}u_{i}^{k}+\sum_{i\in I}v_{i}^{k}} \end{array}$$

Here, we assumed that for each voxel *i* of the image, a softmax output vector $u_{i}\in {\mathbb{R}}^{K}$ with K = # classes is output by the model. Consequently, the ground truth label *v*_*i*_ of the voxel must be encoded as a one-hot vector. Thus, all but one of the terms $u_{i}^{k}v_{i}^{k}$ in the numerator of Equation (2) evaluate to zero for each voxel. The Dice Loss is particularly well-suited for smaller structures, such as the Brainstem or Hippocampus, since it is unaffected by correctly classified background voxels. Other design choices related to the training procedure include the use of stochastic gradient descent with an initial learning rate of 1e^−2^, a Nesterov momentum of 0.99 and weight decay and the use of a scheduler which decays the learning rate via the scheme ${\left(1-\mathrm{epoch}/{\mathrm{epoch}}_{\max}\right)}^{0.9}$.

#### 3.2.4. Architecture of the segmentation model

The ideal network design to handle a patch size of 128 × 128 × 128 is a 3D U-Net consisting of an input block, four downsample blocks, a bottleneck block, five upsample layers, and an output block. The number of convolutional blocks per resolution step was kept fixed at two. Two convolutional blocks were followed by an instance normalization and a Leaky ReLU non-linear activation in a resolution step. Instance normalization was preferred over batch normalization because of the small batch sizes. In the bottleneck layer, the spatial size of the 3D image was reduced to 4 × 4 × 4. The architecture largely follows a “vanilla” variant of the original U-Net, with three main modifications. Firstly, kernels and strides were adapted to 3D images. Secondly, residual connections were used within the convolution blocks of the U-Net. Lastly, deep supervision was utilized (see Zhou et al., 2018). Here, the loss was calculated not only from the final output of the segmentation network and the ground truth label, but by taking outputs from deeper layers with a lower resolution into account as well. To calculate a “deep supervision loss,” these outputs from deeper layers are compared with downsampled versions of the ground truth segmentations. How many of the deeper layers of the U-Net are included in this deep supervision process is a hyperparameter of the network architecture; however, it is not reasonable to include an undersampled output near the bottleneck layer (Isensee et al., 2019). The loss function defined in Equation (3) must be adjusted. Erroneous segmentations coming from the low-resolution outputs of the deeper layers should be given less weight than the final segmentation maps (Isensee et al., 2019):


(3) $$\begin{array}{l} L_{total}=\omega_{final}\cdot L_{final}+\omega_{-1}\cdot L_{-1}+\omega_{-2}\cdot L_{-2}+\ldots \end{array}$$

where ω_−1_ is the weight given to the loss based on the penultimate layer of the U-Net, ω_−2_ is the weight given to the loss based on the layer before that, and so on.

#### 3.2.5. Inference

Performance evaluation on a held-out test set should not be done on patches as in training and validation, but on realistic, full resolution images. A sliding-window approach (Isensee et al., 2018) was used to address the problem of the network architecture being designed to handle inputs of a fixed patch size only. Full resolution images were first divided into overlapping patches of the size of the training data and fed to the model. Output segmentations were then aggregated into a full size segmentation mask by averaging predictions for each voxel. No further processing was carried out on the segmentation output.

### 3.3. Extending the model to perform age prediction

Subject age is an important source of variance in neonatal brain MRI (e.g., Makropoulos et al., 2018a). This is demonstrated by the fact that spatiotemporal probabilistic atlases, which provide weekly or sub-weekly mean images and continuous spatial transformations over time, are very popular in the neonatal literature (Serag et al., 2012; Schuh et al., 2014). It is also an often cited reason for the particular difficulty of neonatal brain segmentation (Makropoulos et al., 2018a). Since metadata that includes subject age is available in the public *dHCP* release, an obvious way around this difficulty would be to provide a model not just with an MR image, but also with the corresponding subject age. However, this would be of little use in practice as information about the post-conceptional age of an infant may not always be available. Instead, we propose extending the segmentation model by introducing an age prediction pathway, terming this new model *AgeU-Net*. In addition to potentially introducing a regularizing effect to the model, accurate age prediction without much added computational effort can be considered useful on its own.

#### 3.3.1. Architecture of extended model

**Figure 3:**
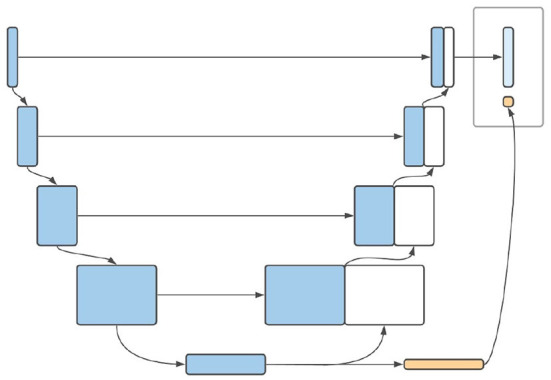
Schematic illustration of the proposed architecture of *AgeU-Net*.

3D CNNs have been successfully used for accurate prediction of brain age in adults based only on MRI scans (Cole et al., 2017; Jónsson et al., 2019; Peng et al., 2021). The encoder path of a 3D U-Net, on the other hand, represents nothing other than a CNN. We therefore suggest using the outputs of the encoder pathway of the U-Net (i.e., the bottleneck layer) as a starting point for age prediction. In the bottleneck layer of the U-Net, the spatial information of the image is maximally condensed. The output of this layer has low spatial resolution with a large number of channels (Ronneberger et al., 2015; Isensee et al., 2019). This resolution could be reduced by further convolutional layers until a resolution of # channels × 1 × 1 × 1 is reached, which in turn can serve as input for a fully-connected layer used for age regression. The result of this additional pathway can be combined with the segmentation map to produce a model output. A schematic diagram of this model can be found in Figure 3. In order for the network to be able to learn the age of the infants from the scans, a second loss ought to be defined. Since the final layer of the age pathway performs a regression task, it is natural to use the mean squared error (MSE). Leaving aside deep supervision, the total loss of a combined prediction can then be defined as:


(4) $$\begin{array}{l} L_{total}=L_{DiceCE}+\alpha L_{MSE} \end{array}$$

where α represents the weighting factor that determines how much the network should prioritize producing accurate segmentations over learning the subject's age. A value of α = 10e^−3^ was determined empirically^2^. For evaluation, all patch-based age predictions generated by the sliding-window approach are accounted for. For each image, not only the segmentation is now evaluated, using a sliding-window-approach, but also the age prediction. For this purpose, the MSE as well as the mean average error (MAE), which is defined as the average absolute value the of deviation, were used.

### 3.4. Label budgeting

The problem of insufficient ground truth labels in medical imaging, especially in relation to neonatal data, is mentioned in the literature with striking frequency (Makropoulos et al., 2014, 2018a; Menze et al., 2014; Devi et al., 2015; Amiri et al., 2019 and others). Since manual labeling of scans by experts is associated with time and financial costs, one can reframe the problem as there being a certain limited label budget. This label budget may contain, for example, a number of 2D scans that ought to be annotated on certain modality and along a certain axis. The question of how this budget can be allocated as efficiently as possible, i.e., “which allocation would lead to the best segmentation performance?” was investigated empirically. We defined the baseline ideal condition as the labeling of whole 3D brain scans. This was compared to different strategies where limited 2D slices to be annotated were distributed over several subjects instead. The extent to which those 2D slices can be distributed over different subjects is called “label dispersion” in this paper and is the first parameter to be investigated. The second one is the axis from which the slices are taken: sagittal, coronal, axial, or at random.

#### 3.4.1. Partially annotated training data

We made use of the segmentation labels available in the public *dHCP* release as pseudo ground truth to investigate different distribution strategies. This was done by *hiding* certain existing labels depending on the factor to be investigated. If the idea of a labeling budget is to be implemented as realistically as possible, then the slices to be annotated must be determined before any kind of transformation (e.g., data augmentation) is applied to the images, and also before the images are cropped to patches. Additionally, simply selecting 2D slices during training and only evaluating on those would not be appropriate. We also decided against training a 2D model on a limited budget of pre-selected 2D labels as this approach would have two serious drawbacks. First, it would be computationally and memory-inefficient to first split the 3D volumes into 2D scans. Second, spatial information would be lost because if a 3D volume is only partially annotated, a segmentation method can at least gather spatial information about voxel intensities from the 3D scans when it also takes the non-annotated slices into account. Instead, we dealt with a partially annotated 3D label, which went through the same pre-processing steps as the corresponding training image. In other words, if the selected 2D slices were individually fed into our model, the model would not have access to spatial connections that exist between the slices and hence may not recognize which structures are nearby or that the slices belonged to the same whole brain. We have therefore made use of partially annotated 3D scans, but ensured that the loss was calculated on the annotated slices only.

The two main factors whose influence on segmentation performance was investigated are label dispersion and slicing axis. Hypothesizing which method might lead to the best overall performance was not trivial, since some of the mediating factors have effects in opposing directions. Some of these effects are:

- *Neglecting spatial information*: If a model is trained solely on selected 2D slices within partially annotated 3D scans, valuable information about the 3D context of the tissues may be lost. For example, the network might be encouraged to learn where a particular structure is present just with respect to one axis, rather than including information about the voxel intensities in the adjacent slices. Additionally, if several slices directly adjacent to each other are missing, the model would not be capable of utilizing information about the surrounding tissues, which is usually readily available in fully annotated scans. Such effects may suggest a better performance with fully annotated brain scans.
- *More diversity in the training data*: If the labels are distributed over many brain scans, a greater diversity of individual subjects can be covered. This could lead to better generalizability and performance.
- *Unnecessary information in marginal areas of the scans*: Even after foreground cropping, the marginal areas of the scans often contain little information, as often only a few of the tissues are represented here. If only selected 2D slices are annotated by brain scan, one could ensure that the marginal areas (e.g., the outermost 10% along each axis) are left out and thus the information density is increased. This effect suggests better performance with partially annotated brain scans.

Additionally, with respect to the slicing method, one could expect that if the slices are taken from an axis that has a significantly lower resolution than the other axes, there may be a small performance dip. In the *dHCP* dataset with images of size 217 × 290 × 290, this would be the sagittal axis. The loss was calculated on the basis of the labeled slices only, as detailed below.

**Figure 4:**
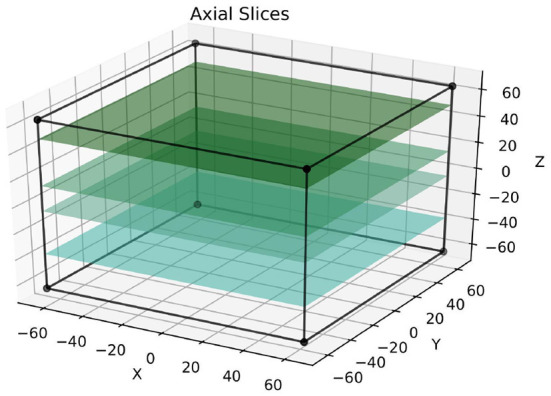
This figure visualizes the extraction of annotated 2D slices from a 3D volume, where colored slices correspond to labeled data. The approach is referred to as *axial* following the terminology used in radiology. The sizes of the cuboids are true to the real training patches.

The labeled slices were fixed for each sample and were defined prior to the training process; Figure 4 depicts the selection of slices in the axial axis. Following this, a mask-like structure was created for each image, which defined the exact parts of the image that are not annotated. Given that there were several data augmentation steps, this was not trivial since the masks had to go through these exact steps. To illustrate why this is important, say an image was mirrored during data augmentation; if the mask does not go through this exact mirroring step as well, the wrong side of the image would ultimately be masked. Finally, during training, the loss for each partially annotated 3D scan was calculated based on the parts of the image that were labeled (i.e., unmasked). Other parts of the images were simply ignored. If this was done differently, for example by replacing the unlabeled regions of the image with zeros, it would have most likely introduced an element of bias into the model. Of course, the above only applies to the training data, and all models were tested on fully annotated 3D scans^3^.

### 3.5. Transfer learning

Transfer learning can potentially be used to alleviate the absence of sufficient ground truth. We investigated the efficiency of this approach in a series of experiments using two datasets constructed from the publicly available *dHCP* release.

#### 3.5.1. Definition of source and target

**Figure 5:**
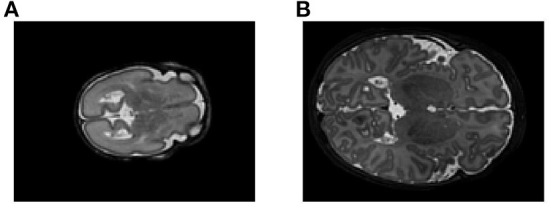
Axial slices of T2-weighted scans of: **(A)** the youngest subject, 26.71 weeks post-conceptional age, vs. **(B)** the oldest subject, 45.14 weeks post-conceptional age in the dataset. The head circumference of the oldest infant on the left is 36 cm, compared to 21 cm of the youngest infant on the right.

In a typical supervised machine learning scenario, one can define the *domain* D={X,P(X)} as a set of a feature space X and the marginal probability distribution of the training samples *X*, *P*(*X*). A *task* T={Y,P(y|x)} can correspondingly be defined as a set consisting of a label space Y and an objective function which we want to predict, usually the conditional probability of a certain training sample belonging to a particular class. Transfer Learning “allows the domains, tasks, and distributions used in training and testing to be different” (Pan and Yang, 2009, p. 2). Transfer learning techniques can be classified into different settings. The *inductive approach* only assumes that the target task is different from the source task (Pan and Yang, 2009); no assumption is made about the two domains. The *dHCP* dataset is particularly well-suited for constructing two disjoint datasets for such an inductive approach, because a broad age spectrum is represented in it. Visual inspection of the two extremes of the dataset—an infant at 26.71 and at 45.14 weeks—confirms that neurophysiological differences are striking, (see Figure 5). On the one hand, this is due to the different stages of brain development (Makropoulos et al., 2018a). The fact that the younger subjects of the dataset are necessarily preterm infants, whose brains are known to differ from those of term infants, likely plays a role as well. A source dataset was therefore constructed from the population of older infants, whereas the target data was composed of the youngest individuals. Distributions of voxel intensities were assumed to differ between these two groups, solely based on differences in head circumference. The same applies to the target predictive functions. Different degrees of myelination in older vs. younger individuals and their effect on T1 signaling mean that voxel intensities in some regions have a different meaning, depending on their group membership. In concrete terms, this means that the probability of a bright voxel belonging to the tissue class “White Matter” is higher for older subjects compared to younger ones. Feature as well as label spaces do not vary between source and target datasets.

#### 3.5.2. Transfer learning strategies

Taking inspiration from Yosinski et al. (2014) and Amiri et al. (2019), six strategies to adapt a baseline model pre-trained on a source dataset of older infant brains to the task of segmenting preterm infant brain MRI were investigated. These strategies differed in the degree to which they incorporated preterm infant training data. The most extreme case was the “No Finetuning” condition; here the pre-trained network was directly evaluated on the preterm test data but the training dataset remained untouched. Four degrees of finetuning were then examined, starting with a condition where only the bottleneck and directly adjacent convolutional layers of the U-net were trained, while all parameters of the outer layers in the expanding and contracting pathways remained fixed. Then, successively in the “Medium” and “Deep” stages, more of the outer layers were included in the training process. In the “Finetuning” condition, all network parameters were updated during training. An additional model was trained exclusively on the 40 images of the preterm infant training dataset.

### 3.6. Technologies used

All experiments were conducted on Nvidia Tesla T4 (16GB RAM) graphics cards and programmed in Python 3.8.10. For the implementation of the pipeline features, we relied on PyTorch and MONAI^4^, an open-source, PyTorch-based framework for deep learning in medical imaging. For data handling, data analysis, and evaluation of the experiments, pandas and scitkit-learn were also used.

## 4. Results

### 4.1. Segmentation pipeline and age prediction

#### 4.1.1. Segmentation pipeline

A first set of experiments was conducted to investigate the performance of the segmentation pipeline for neonatal brain MRI and to establish a performance baseline in segmentation across all age groups for all further experiments. For training, an age-representative subset consisting of 20% of the available 709 complete data units of the *dHCP* dataset was used, corresponding to 142 neonatal brain scans. These data units each consisted of skull-stripped T1- and T2-weighted MRI scans and accompanying (pseudo ground truth) segmentation masks. The 142 data units were again divided into 114 training and 28 validation images. An independent test set was constructed by selecting another 100 separate scans from the total *dHCP* dataset. The training data went through the pre-processing and data augmentation steps described above. The U-Net model was trained on the data over 60 epochs, and experiments were repeated over four runs. Training took an average of 18.6 h. After each epoch, the mean DSC as well as the individual DSC values related to each of the tissue classes on the validation data were determined. It should be noted that the validation data was cropped to the same patch size as the training data and model evaluation was thus carried out directly on the whole patches. In contrast, a sliding window approach was used to evaluate the model on the held-out test set.

**Figure 6:**
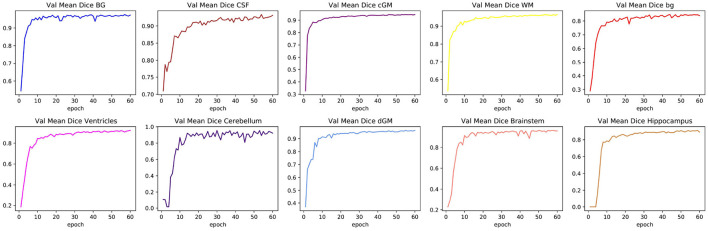
Changes in validation DSC for all physiologically relevant tissues. The figure shows one particular run. The plots illustrate how a large proportion of the learning process took place within the first 20 epochs, and how some tissue classes can reach a plateau more quickly than others.

**Figure 7:**
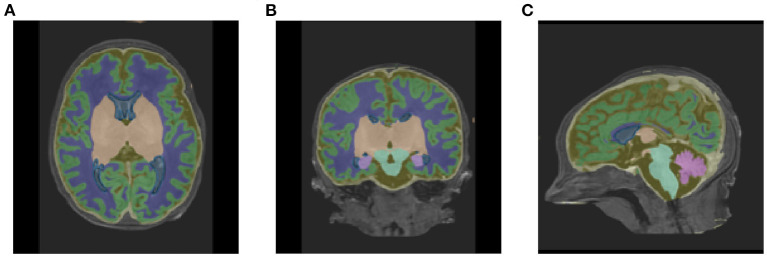
Segmentation outputs in **(A)** axial, **(B)** coronal, and **(C)** sagittal views produced by the baseline pipeline, visualized on the original T1-weighted scan of a subject with a scan age close to the medium of the distribution (38.86 weeks PMA).

**Figure 8:**
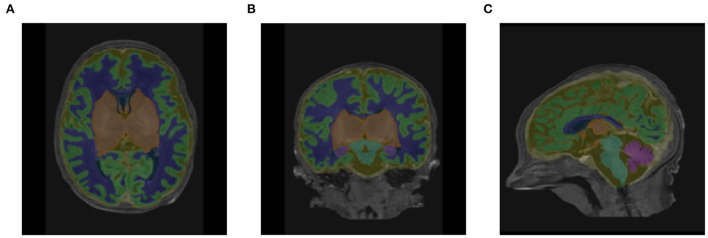
Corresponding labels used in place of ground truth in **(A)** axial, **(B)** coronal, and **(C)** sagittal views for the same subject as shown in Figure 7 (38.86 weeks PMA).

Table 1 shows the mean DSC values averaged over all 10 segmentation classes and over all 4 runs, including two background classes (“Background (outer)” and “Background (inner)”) as well as a mean DSC that only includes the physiologically relevant tissue classes. The table includes those metrics both for the validation dataset and the held-out test set. The best performance achieved by a model on the test set was 0.913 averaged over all classes and 0.917 only based on the physiologically relevant tissues. Table 2 breaks down these results further for the individual tissue classes. Highly accurate results were achieved both on average and in relation to the individual tissues classes. The DSC was at least 0.9 for the majority of the tissue classes and was only around 0.877 for the ventricles in the worst case. The model achieved particularly accurate results in the segmentation of White Matter, Dark Gray Matter, and the Brainstem. Performance seemed to reach a plateau after 30–40 epochs and significantly earlier for most tissue classes. See Figure 6 for a plot of the validation DSC values over epochs, separately for each of the tissue classes. For all tissues, a performance drop compared to the validation data was observed. With the data available, it cannot be clearly disentangled whether this is due to overfitting (since, in each run, the final model was chosen as the one which performed best on the validation data) or due to the fact that the validation data was evaluated in patches of the same size as the training data, while the test data was evaluated with a sliding window approach. Variations in the mean DSC were low, which prompted us to keep the number of repeated runs lower in the following sets of experiments. An example segmentation of an infant brain with an age close to the center of the overall age distribution is provided in Figure 7, next to the corresponding pseudo ground truth label (Figure 8).

**Table 1:** Mean DSC for all nine tissue regions, averaged over four runs, computed on both the validation set and the held-out test set.

	**Validation set**		**Test set**	
	**Mean**	**Mean DSC**	**Mean**	**Mean DSC**
	**DSC**	**(no BG)**	**DSC**	**(no BG)**
Mean DSC	0.934 ± 4e − 4	0.943 ± 4e − 4	0.913 ± 3e − 3	0.917 ± 3e − 3

The two types of background classes, Background (outer) and Background (inner), were excluded from Mean Dice (no BG) which is highlighted.

**Table 2:** DSC values for each of the eight physiologically relevant tissue classes, averaged over four runs, computed on the validation set and the held-out test set.

	**CSF**	**cGM**	**WM**	**Ventr**.	**Cereb**.	**dGM**	**BS**	**Hippoc**.
VAL DSC	0.925	0.943	0.961	0.907	0.950	0.960	0.961	0.903
TEST DSC	0.919	0.926	0.932	0.903	0.895	0.940	0.940	0.877

#### 4.1.2. Age prediction

**Figure 9:**
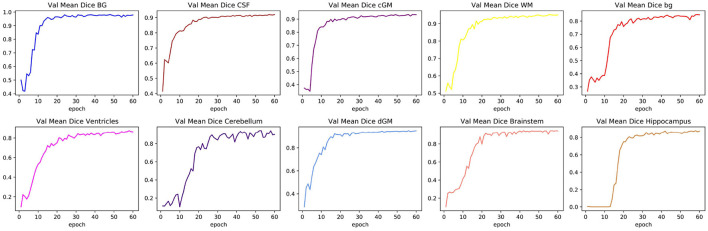
Changes in validation DSC of *AgeU-Net* on all physiologically relevant tissues. A large proportion of the learning process took place within the first 20 epoch, similar to the initial model.

Two questions were of interest in this set of experiments, the first being whether the *segmentation* performance on the held-out test set could be improved by adding an age prediction pathway. The second question concerns whether the age prediction pathway could produce accurate results on its own. The same datasets were used as training and test sets as for the baseline model. The choice of a suitable learning rate turned out to be a difficult problem: if this was chosen in a similar fashion compared to the baseline model, the MSE loss of the age prediction approached infinity after just a few epochs. Significantly smaller initial learning rates helped the age prediction to achieve better results, but slowed down the learning process of the segmentation or stopped it from learning completely. A medium learning rate of 10e^−3^ was chosen to accommodate both parts of the network. The experiment was repeated twice, with the model being trained over 60 epochs per run. Figure 9 shows the validation DSC values on all physiologically relevant tissue classes, plotted over epochs. AgeU-Net still performs reasonably well on the test set, but worse than the baseline. If background classes are excluded, the model achieves a DSC of 0.866. The results concerning the age prediction looked initially tempting: At the end of the 60 epochs, the MSE loss reached a value of 2.45, corresponding to an average deviation of 1.57 weeks. With a range of 18.43 weeks in the total data set, such an accurate prediction of age could be useful as a by-product of a still reasonably accurate segmentation. Unfortunately, when recording the MSE as well as the MAE on the test set, similar results could not be achieved. Closer inspection of the outputs revealed that most of the predictions were in the upper range, close to the average of the age distribution. It can thus be assumed that the age pathway primarily learned to estimate the mean value of the age distribution. The desired regularizing effect on the segmentation method did not occur.

### 4.2. Label budgeting

Five alternative ways of distributing a limited amount of annotated labels over training data were investigated. Four of these alternatives belong to the high-label-dispersion condition. They differ from each other with respect to their slicing axis, i.e., sagittal, coronal, axial, or random. In each of these four conditions, the proportion of selected slices per brain was 33%. In other words, two-thirds of the slices along the respective axis were hidden from the model during training. To enable a fair comparison, the number of data units in the training dataset was tripled compared to the first set of experiments. This was done to ensure that the models trained in the high-label-dispersion condition saw the same total number of annotated 2D slices; thus eliminating a potentially confounding factor of the results. Slices were not selected from the outermost regions of the image, since these usually do not contain much information, as many of the tissues are not yet visible. For each of the four conditions, a new model was trained. The basic configuration of the pipeline remained identical to the baseline model, except for the functionalities that directly affected label hiding and the selection of annotated slices. The number of training epochs was reduced to 20.

Results of those four runs, as well as those of a baseline model after 20 epochs, are compiled in Table 3. Performance was consistently higher when annotated slices were distributed over a larger number of scans, provided the number of training epochs was kept constant. Compared to the baseline model, the drop in performance between validation and test set was less pronounced. The high-dispersion models (random, sagittal, coronal, and axial) hardly showed any deterioration in performance and generalized very well to unseen data, which could be due to a higher diversity in the training data. Related to this, the performance on the validation set was also found to stabilize after a much shorter period of around 13 epochs. Notably, the performance of the model trained in the sagittal condition, where the slices were selected along the axis with the lowest resolution, was the worst of all models except the baseline.

**Table 3:** Mean DSC values of the four budgeting conditions.

	**Validation set**		**Test set**	
	**Mean**	**Mean DSC**	**Mean**	**Mean DSC**
	**DSC**	**(no BG)**	**DSC**	**(no BG)**
Random	0.913	0.913	0.910	0.914
Sagittal	0.912	0.912	0.900	0.907
Coronal	0.911	0.910	0.913	0.917
Axial	0.913	0.912	0.905	0.908
Full (20 epochs)	0.908	0.909	0.852	0.849

In the last row, the scores of the model trained on fully annotated brains (baseline) are shown. To facilitate comparison, the scores given in this table do not reflect the performance of the baseline model after 60 epochs (as in Table 1). Instead, the best model on the validation set after 20 epochs was selected and again evaluated on the test set. The highlighted column indicates test set results on the tissues excluding the background.

Even though the DSC values of the models in the “high label dispersion” conditions were better than those of the baseline model in a comparable setting, the number of epochs and segmentation metrics are not the only relevant metrics to take in consideration when evaluating the performance of a pipeline. In practice, a possibly decisive factor when choosing a framework is training time. The average training time of the four runs in the high-dispersion condition was 27 h and 42 min, with a standard deviation of 4 h and 20 min. This represents a 49% increase in training time compared to a baseline model trained over a total of 60 epochs, while not producing better segmentation results.

### 4.3. Transfer learning on preterm and term infants

**Figure 10:**
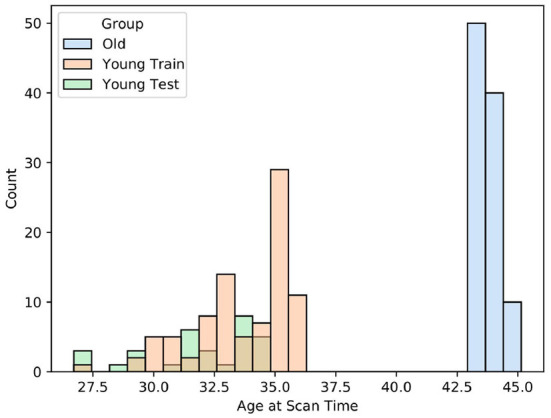
This figure depicts the age distributions of the source data *Old* and the target data *Young Train*. Additionally, the age distribution of the held-out test set for the young population *Young Test* is shown. We carried out a *t*-test which determined that differences in age between *Young Train* and *Young Test* were unlikely to be significant (*t* = −0.351, *p* = 0.7267), so the held-out test set can be assumed to be representative of the population.

The source dataset made up of term infants was constructed by splitting the 100 oldest subjects from the full *dHCP* dataset. The mean scan age in this group was 43.70 weeks post-conceptional age, with a standard deviation of 0.47 weeks (i.e., about 3 days). The oldest subject in this dataset was 45.14 weeks at scan age (see Figure 5); the youngest was 42 weeks. For the construction of the target dataset, the 120 youngest subjects were selected from the full dataset. The mean age in this group was 33.19 weeks with subjects ranging from 26.71 to 35.71 weeks post-conceptional age. Of these 120 preterm subjects, another 40 were randomly selected as the target training set. The remaining 80 scans were used as an independent test set. The training data set was kept small to simulate the problem of missing data in the target domain. The age distributions of all three datasets can be found in Figure 10. It is important to note that because of the negatively skewed age distribution of the *dHCP* dataset, the range of scan ages in the target dataset was larger, possibly making the target task more difficult.

**Figure 11:**
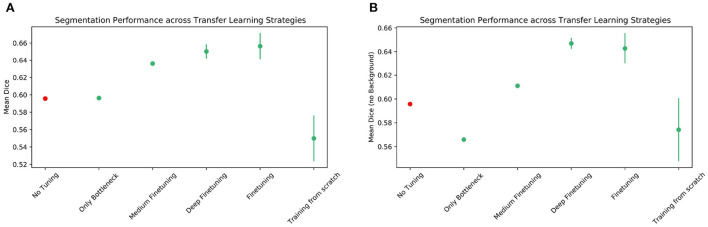
re contains two plots detailing the segmentation performance, averaged over four runs each, of the transfer learning strategies presented. **(A)** shows the mean DSC across all classes, while in **(B)** background classes are excluded. Added error bars of length “standard deviation” mark the variability of the scores. The condition *No Tuning* is marked in a different color to emphasize that only one run was tested.

We started out by training a baseline model on 90 brain scans of the source dataset, i.e., the dataset of term infants, for a maximum of 20 epochs. A small number of 10 scans was reserved for a validation step to carry out a rough quality control. The mean DSC after those 20 epochs was 0.897, which is in line with previous experiments, given the fact that the size of the training set was reduced. In the next step, six transfer learning strategies were examined. In the “no fine-tuning” condition, the pre-trained model was directly evaluated on the held-out test set of preterm infants. We decided to pivot from the fine-tuning strategies described by Amiri et al. (2019). Since the bottleneck layer of the U-Net contains the most condensed information about an image, we successively included more and more layers in the training, from the inside to the outside of the U-Net, instead of restricting to the contracting/expanding path. In the “only bottleneck” or “shallow” condition, all weights except those of the bottleneck and the directly adjacent layers were fixed. In the “medium” condition, one further U-Net block of the encoder and the decoder path was added to the training. In the “deep” condition, two more downsampling and upsampling blocks were added, while in the “fine-tuning” condition, all weights were updated during training. Another baseline U-Net was trained ‘from scratch' for a total of 30 epochs on the preterm infant training data. Results of these experiments are compiled in Table 4 and Figure 11, and Bonferroni-corrected *p*-values are shown in Tables 5, 6.

**Table 4:** This table displays the DSC values of all transfer learning strategies examined in the experiments, for each of the eight physiologically relevant tissue classes.

	**CSF**	**cGM**	**WM**	**Ventr**.	**Cereb**.	**dGM**	**BS**	**Hippoc**.
No tuning	0.778	0.723	0.712	0.744	0.010	0.327	0.765	0.549
Shallow	0.780	0.712	0.617	0.733	0.012	0.361	0.758	0.553
Medium	0.791	0.718	0.604	0.693	0.065	0.604	0.781	0.632
Deep	0.808	0.757	0.446	0.689	0.201	0.801	0.794	0.680
FT	0.811	0.816	0.464	0.598	0.185	0.818	0.800	0.650
New	0.791	0.689	0.201	0.671	0.463	0.739	0.700	0.339

For each class, the best performing model was marked in green. Scores below 0.5 were marked in orange.

**Table 5:** Bonferroni-corrected *p*-values of independent *t*-tests for each pair of transfer learning strategies.

	**Only bottleneck**	**Medium finetuning**	**Deep finetuning**	**Finetuning**	**Training from scratch**
Only bottleneck	*	1.67e − 7	2.56e − 4	4.40e − 3	2.23e − 1
Medium finetuning	*	*	2.26e − 1	5.67e − 1	1.30e − 2
Deep fine-tuning	*	*	*	1.0	7.29e − 3
Finetuning	*	*	*	*	8.84e − 3
Training from scratch	*	*	*	*	*

The two background classes were *not* taken into account in the calculations. All tests were two-sided, with an overall alpha level of α = 0.05. Mean differences where *p* < 0.05 are highlighted in green.

**Table 6:** Bonferroni-corrected *p*-values of independent *t*-tests for each pair of transfer learning strategies.

	**Only bottleneck**	**Medium finetuning**	**Deep finetuning**	**Finetuning**	**Training from scratch**
Only bottleneck	*	1.04e − 7	1.06e − 6	4.75e − 4	1.0
Medium finetuning	*	*	1.27e − 4	5.26e − 2	5.21e − 1
Deep fine-tuning	*	*	*	1.0	3.33e − 2
Finetuning	*	*	*	*	6.76e − 2
Training from scratch	*	*	*	*	*

All classes were taken into account in the calculations. All tests were two-sided, with an overall alpha level of α = 0.05. Mean differences where *p* < 0.05 are highlighted in green.

When comparing the overall performance of the different transfer learning strategies, we observed two main patterns. First, strategies that included more parameters in the training (i.e., deeper fine-tuning) were superior. Fine-tuning only the bottleneck and adjacent layer was clearly inferior to all other strategies, but the performance did not seem to depend linearly on the number of parameters included in the training. For instance, the difference between the shallow and medium fine-tuning conditions was only the addition of two more blocks. Yet, in the deep fine-tuning condition, four new blocks were included in the training. Despite this, the performance gain between the shallow and medium conditions was even *higher* than that between medium and deep fine-tuning (Figure 11). The second pattern we observed was that including more parameters in the training resulted in greater variability in performance.

Despite the above, our results suggest that the models did not benefit at all from transfer learning when segmenting the Cerebellum. In fact, the baseline model trained from scratch on preterm infant data outperformed all other strategies on this tissue class. However, the opposite was true for White Matter, where the pre-trained model showed the best performance without any further adjustments. Despite showing remarkable differences, none of the strategies we investigated appear to be up to the difficult task of segmenting the scans of very preterm infants, and some tissues such as the Cerebellum and Hippocampus were particularly hard to segment in general (see Table 4).

In summary, the transfer learning experiments yielded three main findings on the data we used. First, if fine-tuning is required, the effects of increasing the number of parameters included in the training may not be linear, and medium fine-tuning may be enough to achieve similar results to those obtained when fine-tuning the entire model. Second, the higher the number of parameters to be included in the training, the higher the variance of the results. Finally, the question of which strategy is most effective depends on the individual tissue classes under consideration, possibly due to specific biological differences between preterm and term infants.

## 5. Discussion and future work

The first goal of this paper was to present an accurate deep learning-based segmentation pipeline for neonatal brain MRI. For this purpose, publicly available data from the *dHCP* was used, which contains a large number of infant brain scans over a wide age range, as well as highly accurate segmentation labels that we used as pseudo ground truth labels. A U-Net-based architecture was specifically tailored to this data. Pre-processing, data augmentation, training hyperparameters, and network topology were optimized to achieve excellent segmentation performance on physiologically relevant tissues. As a final result of the pipeline, sample segmentations can be found in Figure 7. In addition, we proposed an extension to this baseline U-Net model that enables the network to predict age. Although the regularization effect we had hoped for did not manifest in our experiments, the idea of an age-predicting U-Net is an interesting approach. The problem of different optimal learning rates for the segmentation and regression task could possibly be solved by layer-specific learning rates in future experiments. Alternatively, it could be investigated whether an increasing schedule of the MSE weighting parameter α leads to better results, i.e., by taking the MSE loss into account more strongly at the end of the training, or by separating training for segmentation and regression entirely.

An important step was to identify reliable ground truth annotations that can guide the development of the segmentation pipeline. The gold standard in medical image segmentation is manual delineation (Gousias et al., 2012), therefore, developing a robust deep learning-based pipeline would conventionally require large amounts of ground truth labels to be annotated manually by experienced annotators. We avoided this by utilizing labels that were generated by the *dHCP* initiative using an automated software pipeline (not deep learning-based), and are publicly available as part of the *Third Data Release*. The software pipeline that had generated the labels was specifically designed for neonatal brain MRI data based on the DrawEM framework (Makropoulos et al., 2014) and was discussed by Makropoulos et al. (2018b). Noteworthily, the *dHCP* structural data and anatomical segmentation had undergone a quality assurance process detailed in their release notes, which identified small areas of common segmentation inaccuracies; however, we did not carry out manual refinements on the labels we used.

The second goal of this paper was to investigate how to deal with a lack of sufficient ground truth labels as efficiently as possible. Two independent approaches were pursued. The first was the question of whether better performance could be achieved through more efficient label budgeting, and our results suggest that this may well be the case. Models trained on a larger variety of partially annotated brains outperformed a model trained on a smaller variety of fully annotated brains, provided other conditions (number of epochs, number of annotated slices seen during training) were kept constant. At the same time, significant increases in training time call the benefits into question. Due to time and computational constraints, we had to limit ourselves to two conditions of “label dispersion.” With more resources, the effect of this factor could be investigated more systematically in future work, for instance, by examining even more extreme cases of dispersion, where only single 2D slices are taken per subject. Although we covered all three possible slicing axes in our experiments, it would be interesting to see whether the effect of poorer performance when choosing a lower-resolution axis can be replicated with other datasets, especially datasets where differences in axis resolutions are more pronounced. There may be other variables that influence optimal label budget distribution, e.g., whether labels are extracted from the center of the brain or fromregions where there is a high probability of a particular tissue being present.

Our second approach to address the problem of a ground truth bottleneck was based on transfer learning. Using two datasets constructed from the *dHCP* dataset, we investigated how a model pre-trained on older term infants could be optimally adapted to the task of segmenting preterm infant brain MRI. Our results confirm Yosinski et al. (2014) and Amiri et al. (2019)'s findings that a fine-tuned model is the best choice overall. Especially if the target dataset is small, this model cannot be outperformed by a model trained from scratch. At the same time, it does not seem necessary to tune all layers of a network to achieve similar results, since performance gains do not depend linearly on the number of parameters included in the training process. Most importantly, however, whether a transfer of network knowledge can succeed seems to depend on the tissue class and the specific relation between source and target data and might be hard to predict for a layperson. While pre-training and fine-tuning had clear positive effects on segmentation performance for some tissue classes, in other cases it seemed to prime the model in an unhelpful way. Future work could investigate whether certain heuristics about the relationship between source/target data and the best transfer strategy might be established. In addition to this, investigating the utility of models pre-trained on other types of medical images (e.g., using RadImageNet, Mei et al., 2022) may spawn interesting future research.

A limitation to our work is that quantitative evaluation relied solely on DSC, which is considered the *de facto* evaluation metric by the medical image computing community for segmentation tasks. Despite its widespread use in research studies, DSC does not take into account the spatial context within an image and can therefore be of limited utility in clinical settings. Complementary metrics ought to be explored in the future, namely 95th Percentile Hausdorff Distance (HD95), which can provide a relatively robust estimate of how well the boundaries of the structures match the model's output.

Finally, we would like to draw the reader's attention to an inherent tension between the theoretical background and our experiments. The work of Isensee et al. (2018) calls into question whether transfer learning can be of use at all; even if domain and task of two datasets are very similar, properties such as the patch size of the dataset can change the dataset fingerprint. The optimal network topology and other pipeline parameters might change accordingly. The nnU-Net framework makes network architecture decisions based primarily on surface properties of the datasets. Whether the input consists of MRI scans, X-ray scans, or even natural images is irrelevant for the proposed network architecture. On the other hand, properties such as image resolution play only a minor role in the mathematical formulation of the transfer learning problem, since they could be resized in principle. The transfer learning task we have investigated here satisfies the conditions of similarity both in terms of domain content (i.e., the distribution of voxel intensities in both the domain of the older and the domain of the younger subjects can be assumed to be similar) and in terms of fingerprint-relevant surface properties. A question arises as to how the benefit of transfer learning would be weighed if the target dataset had a completely different fingerprint. It is quite possible that the effect of a suboptimal network architecture would cancel out the positive effect of transfer learning, and that an optimally configured network trained on a very small dataset would outperform such a fine-tuned network. Investigating this offers exciting new possibilities for future research.

## Data availability statement

Publicly available datasets were analyzed in this study. This data can be found here: https://biomedia.github.io/dHCP-release-notes/.

## Author contributions

LR and AF conceptualized the project. LR conducted the literature review, designed the deep learning pipeline, implemented the software, carried out the experiments, wrote, and edited the initial manuscript draft. AF supervised the project, advised on methodology and experimental design, revised, and edited the manuscript. Both authors critically reviewed and approved the final manuscript.

## Funding

AF is supported by the UK Research and Innovation Centre for Doctoral Training in Artificial Intelligence for Healthcare under Grant EP/S023283/1.

## Conflict of interest

The authors declare that the research was conducted in the absence of any commercial or financial relationships that could be construed as a potential conflict of interest.

## Publisher's note

All claims expressed in this article are solely those of the authors and do not necessarily represent those of their affiliated organizations, or those of the publisher, the editors and the reviewers. Any product that may be evaluated in this article, or claim that may be made by its manufacturer, is not guaranteed or endorsed by the publisher.

## Footnotes

^1^ https://github.com/richterleo/Neonatal_Brain_Segmentation

^2^It should be noted that MSE tends to reach values larger by powers of ten than the combined dice/crossentropy loss.

^3^The authors would like to emphasize that complete sets of labels were available for all training samples when developing the pipeline, and that labels were only artificially removed when carrying out the label budgeting experiments.

^4^ https://monai.io
